# The Role of Heparan Sulfate and Neuropilin 2 in VEGFA Signaling in Human Endothelial Tip Cells and Non-Tip Cells during Angiogenesis In Vitro

**DOI:** 10.3390/cells10040926

**Published:** 2021-04-16

**Authors:** Marchien G. Dallinga, Yasmin I. Habani, Alinda W. M. Schimmel, Geesje M. Dallinga-Thie, Cornelis J. F. van Noorden, Ingeborg Klaassen, Reinier O. Schlingemann

**Affiliations:** 1 Ocular Angiogenesis Group, Departments of Ophthalmology and Medical Biology, Amsterdam Cardiovascular Sciences, Cancer Center Amsterdam, Amsterdam UMC, University of Amsterdam, Meibergdreef 15, 1105 AZ Amsterdam, The Netherlands; m.g.dallinga@amsterdamumc.nl (M.G.D.); y.i.habani@amsterdamumc.nl (Y.I.H.); c.j.vannoorden@amsterdamumc.nl (C.J.F.v.N.); r.o.schlingemann@amsterdamumc.nl (R.O.S.); 2 Department of Experimental Vascular Medicine, Amsterdam UMC, University of Amsterdam, Meibergdreef 15, 1105 AZ Amsterdam, The Netherlands; a.w.schimmel@amsterdamumc.nl (A.W.M.S.); g.m.dallinga@amsterdamumc.nl (G.M.D.-T.); 3 Department of Genetic Toxicology and Cancer Biology, National Institute of Biology, Večna pot 111, 1000 Ljubljana, Slovenia; 4 Department of Ophthalmology, University of Lausanne, Jules Gonin Eye Hospital, Fondation Asile des Aveugles, Avenue de France 15, 1004 Lausanne, Switzerland

**Keywords:** endothelial cells, angiogenesis, VEGFA, tip cells, SULF2, NRP2, HSPG

## Abstract

During angiogenesis, vascular endothelial growth factor A (VEGFA) regulates endothelial cell (EC) survival, tip cell formation, and stalk cell proliferation via VEGF receptor 2 (VEGFR2). VEGFR2 can interact with VEGFR2 co-receptors such as heparan sulfate proteoglycans (HSPGs) and neuropilin 2 (NRP2), but the exact roles of these co-receptors, or of sulfatase 2 (SULF2), an enzyme that removes sulfate groups from HSPGs and inhibits HSPG-mediated uptake of very low density lipoprotein (VLDL), in angiogenesis and tip cell biology are unknown. In the present study, we investigated whether the modulation of binding of VEGFA to VEGFR2 by knockdown of *SULF2* or *NRP2* affects sprouting angiogenesis, tip cell formation, proliferation of non-tip cells, and EC survival, or uptake of VLDL. To this end, we employed VEGFA splice variant 121, which lacks an HSPG binding domain, and VEGFA splice variant 165, which does have this domain, in in vitro models of angiogenic tip cells and vascular sprouting. We conclude that VEGFA_165_ and VEGFA_121_ have similar inducing effects on tip cells and sprouting in vitro, and that the binding of VEGFA_165_ to HSPGs in the extracellular matrix does not seem to play a role, as knockdown of *SULF2* did not alter these effects. Co-binding of NRP2 appears to regulate VEGFA–VEGFR2-induced sprout initiation, but not tip cell formation. Finally, as the addition of VLDL increased sprout formation but not tip cell formation, and as VLDL uptake was limited to non-tip cells, our findings suggest that VLDL plays a role in sprout formation by providing biomass for stalk cell proliferation.

## 1. Introduction

Sprouting angiogenesis is the complex process of blood vessel growth from the existing vasculature. It is characterized by differentiation of ECs into subtypes with distinct functions: tip cells are non-proliferating and highly migratory and lead the growing vessel sprout towards a source of growth factors. Tip cells suppress the tip cell phenotype in adjacent stalk cells that are proliferating and start forming a lumen, and further down the sprout are phalanx cells that initiate vessel maturation [1,2]. VEGFA is the main initiator of sprouting angiogenesis [2]. The main receptor for angiogenic signaling by VEGFA is VEGFR2, and its stimulation induces tip cell migration, expression of tip cell genes, proliferation of stalk cells, and EC survival [2,3]. Binding of VEGFA to VEGFR2 is affected by a number of factors, which include binding to extracellular matrix (ECM) components such as HSPGs, or simultaneous binding of VEGFA to one of its NRP co-receptors (NRP1 or NRP2) [4,5,6].

HSPGs are present in the ECM, in particular in the basal lamina [7]. Binding of VEGFA to the HSPGs in the ECM is essential for vascular network development. This is illustrated by the lack of complexity in vascular networks in mice expressing only VEGFA splice variant 121 (VEGFA_121_), which lacks the heparan sulfate (HS)-binding domain of the most common VEGFA splice variant VEGFA_165_ [8]. The importance of VEGFA binding to proteins in the ECM has also been demonstrated using in silico modeling [9], which showed that vascular networks, in which an ECM-binding signal was present, were more stable over time, and that binding of VEGFA to the ECM allowed cells to elongate towards a source of VEGFA [9]. HSPGs are also located in the glycocalyx on the apical membrane domain of ECs, including human umbilical vein endothelial cells (HUVECs) in vitro. The glycocalyx is a negatively charged, organized mesh of membranous glycoproteins, proteoglycans, and glycosaminoglycans [10,11,12] which affects internalization of VEGFR2 after VEGFA binding, thus modulating VEGF signaling outcome [13].

The specific types of sulfate groups in HS molecules are important for the degree of binding of VEGFA_165_. It has been shown that ECs surrounded by HSPGs in the glycocalyx that selectively contain 6-O sulfate groups on their HS molecules are less angiogenic and that binding to and subsequent phosphorylation of VEGFR2 by VEGFA_165_ are reduced [14]. Moreover, zebrafish with genetic defects in specific 6-O sulfotransferases have impaired vascular development [15]. Sulfatase 2 (SULF2) is an extracellular enzyme that selectively removes 6-O sulfate groups from HS chains on HSPG molecules [16]. An important role of SULF2 in post-prandial regulation of serum triglycerides has been described, whereas a role of SULF2 in angiogenesis by modifying VEGFA binding to HSPGs has been hypothesized [17], but not reported as far as we know. It has also been shown that SULF2 inhibits the HSPG-mediated uptake of very low density lipoprotein (VLDL) by ECs [18]. This may affect angiogenesis, since VLDL cargo is used for biosynthesis by proliferating cells. Moreover, VLDL activates several pro-angiogenic pathways such as those of the phosphatidylinositol 3-kinase (PI3K) and peroxisome proliferator-activated receptor (PPAR)-γ pathways [19,20,21,22,23]. Taken together, the literature suggests that HSPGs play a role in regulating angiogenesis, but the exact mechanisms and the possible differential regulation by HSPGs of tip cells and non-tip ECs remain unexplored.

In addition to binding to HSPGs, binding to VEGFR2 co-receptors may also affect the outcome of VEGFA signaling in specific subtypes of ECs during angiogenesis. Since microarray data showed that the mRNA expression of *NRP2* was significantly higher in CD34^+^ tip cells than in CD34^-^ non-tip cells in an in vitro model of tip cells [24], NRP2 may have differential roles in VEGFA signaling in these endothelial phenotypes.

Therefore, we investigated whether modulation of binding of VEGFA to VEGFR2 by knockdown of *SULF2* or *NRP2* affects sprouting angiogenesis, tip cell formation, proliferation of non-tip cells, EC survival, and uptake of VLDL, employing in vitro models of angiogenic tip cells and vascular sprouting.

## 2. Materials and Methods

### 2.1. Cell Cultures

Primary HUVECs were isolated from umbilical cords (obtained from the Department of Gynecology, Amsterdam University Medical Center (Amsterdam UMC), location Academic Medical Center (AMC), Amsterdam, The Netherlands), as described previously [25]. Subjects gave informed consent for the use of tissues and/or serum, and samples were stored anonymously. HUVECs were cultured in M199 basal medium (Gibco, Grand Island, NY, USA) supplemented with 10% heat-inactivated human serum (obtained from the Department of Oncology, Amsterdam UMC, location AMC, Amsterdam, The Netherlands), 10% fetal bovine serum (Gibco), and 1% penicillin-streptomycin-glutamine (Gibco). HUVEC cultures were incubated with antibodies directed against CD31/PECAM-1 (1:100; eBioscience, Vienna, Austria) to check the purity of the ECs. HUVECs were cultured in 2% gelatin-coated (Millipore, Amsterdam, The Netherlands) T75 culture flasks at 37 °C and 5% CO_2_. Experiments were performed with confluent HUVECs at passage 3 of at least three donors. HUVECs were treated for 24 h with VEGFA_165_ (R&D Systems, Minneapolis, MN, USA) or VEGFA_121_ (Prospec, Rehovot, Israel) at a final concentration of 25 ng/mL and insulin-like growth factor 1 (IGF1; Prospec) at a final concentration of 50 ng/mL.

### 2.2. Flow Cytometric Analysis

Cell suspensions were obtained after treatment of adherent HUVEC monolayers with TrypLE (Gibco). Cells were fixed in 2% paraformaldehyde in PBS for 15 min at room temperature and incubated with anti-CD34-phycoerythrin antibody (anti-CD34-PE; clone QBend-10, Thermo Fischer Scientific, Waltham, MA, USA), without permeabilization of the cells, to detect CD34^+^ tip cells. Cells were analyzed using a FACSCalibur (Becton Dickinson, Franklin Lakes, NJ, USA) and FlowJo 6.4.7 software (Tree Star, San Carlos, CA, USA). Non-stained, non-treated cells were used as negative controls. For each experiment, HUVECs of different donors were used, all with various percentages of CD34^+^ cells (~3–30%). Cell proliferation was assessed by measuring incorporation of 5-ethynyl-2*’*-deoxyuridine (EdU) and propidium iodide (PI) using flow cytometry, following the manufacturer’s instructions (Molecular Probes, Eugene, OR, USA).

### 2.3. Apoptosis Assay

Apoptotic cell death in CD34^-^ and CD34^+^ cells was determined at 72 h after siRNA transfection. Cells were stained using FITC-conjugated anti-annexin V (Life Technologies, Eugene, OR, USA) and anti-CD34-PE (Thermo Scientific), according to the manufacturer’s instructions. The apoptotic fraction of CD34^-^ and CD34^+^ cells was detected by flow cytometry using a FACSCalibur (Beckton Dickinson) and analyzed using FlowJo 6.4.7 software (Tree Star).

### 2.4. Spheroid-Based In Vitro Angiogenesis Assay

Seven hundred and fifty cells per spheroid were seeded in methylcellulose-containing medium (Sigma–Aldrich, Buchs, Switzerland) in the presence of 2% human serum to allow spheroid formation using the hanging drop method [26,27]. After 18 h, the spheroids were embedded in collagen gels containing insulin-like growth factor 1 (IGF1) (50 ng/mL) and when indicated, VEGFA_165_ (25 ng/mL), VEGFA_121_ (25 ng/mL), or VLDL (25 µg/mL), and were allowed to sprout for 24 h. Phase-contrast images were taken using an inverted microscope (Leica Microsystems, Mannheim, Germany), and the number of sprouts and average sprout length per spheroid were analyzed by manual determination of the distance between the microvessel and the tip cell sprouting furthest into the gel using the Neuron-J plug-in package for Image-J software [28]. The experiments were performed in spheroids of at least three HUVEC donors, and at least 10 spheroids per condition were analyzed in each experiment. For siRNA experiments, HUVECs were transfected with siRNA as described below at 48 h before spheroids were prepared.

### 2.5. SiRNA Knockdown

HUVECs were transfected with 25 nM *SULF2* or *NRP2* siRNA or a non-targeting control siRNA (Dharmacon, Lafayette, CO, USA), in the presence of 2.5 µg/mL Dharmafect 1 (Dharmacon). The cells were transfected for 6 h using the reversed transfection method as described by the manufacturer. The transfection efficiency was verified by measuring mRNA levels of the target genes and considered acceptable when expression was reduced by at least 70% after 72 h.

### 2.6. VLDL Isolation and Labeling

VLDL was isolated from human plasma using gradient ultracentrifugation [29]. VLDL-Apo B was analyzed in the VLDL fraction using a nefelometric assay (Diasys, Wixom, MI, USA) on a Selectra system (Sopachem, Ochten, The Netherlands). One ml VLDL solution was labeled with DyLight 680 at a 1:10 molecular weight ratio according to the manufacturer’s protocol (Thermo Fischer Scientific) and dialyzed extensively and stored at 4 °C.

### 2.7. VLDL Uptake

Cells were treated with siNT or si*SULF2* and cultured on gelatin-coated coverslips (Thermo Fischer Scientific) for 72 h before treatment with Dylight-680-labeled VLDL (2.5 µg/mL) for 120 min in the presence or absence of heparanase (7.5 mU/mL Sigma–Aldrich). Heparanase hydrolyses HS selectively, whereas heparins are poor substrates for heparanases [7]. Cells were fixed in freshly prepared 4% paraformaldehyde in PBS for 15 min at room temperature, and then nonspecific binding was blocked and cells were permeabilized in PBS containing 10% bovine serum albumin (BSA; Sigma–Aldrich) and 0.5% Triton X-100 (Sigma–Aldrich) for 1 h at room temperature. Next, cells were incubated with a monoclonal primary antibody against CD34 (clone MD34.2; Sanquin, Amsterdam, The Netherlands) and a rabbit anti-early endosome antibody (EEA1, 1:500 dilution; Thermo Fischer Scientific) for 2 h and a secondary anti-mouse Alexa488-conjugated antibody and anti-rabbit Alexa568-conjugated antibody (Life Technologies, Carlsbad, CA, USA) for 1 h. Cells were mounted in Vectashield containing DAPI (Vector Laboratories, Burlingame, CA, USA). Images were captured using a Leica confocal microscope (SP8; Leica Microsystems, Wetzlar, Germany), using a 63× objective. Quantification was performed using Matlab (MathWorks B.V., Eindhoven, The Netherlands), and images of EEA1-positive endosomes were compared with images of VLDL staining to assess endosomal uptake of VLDL using a custom script. Cells were analyzed individually and at least seven cells per condition were measured in each experiment.

### 2.8. Immunohistochemistry and Quantification of Protein Levels

Immunohistochemistry was performed as described above, with some changes. Cells were fixed in freshly prepared 2% paraformaldehyde in Hank’s balanced salt solution (HBSS) for 15 min at room temperature; then, nonspecific binding was blocked with HBSS containing 10% normal donkey serum (017-000-121, Jackson Immunology, Cambridgeshire, UK) and 0.1% Triton X-100 for 1 h at room temperature. Next, cells were incubated with anti-VEGFR2 (AF357, 1:100) or anti-NRP2 (AF2215, 1:100), both from R&D Systems, anti-VE-cadherin (361900, Invitrogen, 1:100), and anti-CD34 (Sanquin, 1:100) diluted in primary antibody diluent (APG500, Scytek, Logan, UT, USA) for 1 h at room temperature. The secondary donkey anti-rabbit Alexa647 Plus, donkey anti-mouse Alexa488 Plus (both from Invitrogen, 1:1000) or donkey anti-goat Cy3 conjugated antibody (1:200, Jackson Immunology) were also diluted in antibody diluent and incubated for 1 h at room temperature. Cells were mounted in Vectashield containing DAPI. Images were recorded using a confocal laser scanning microscope (SP8; Leica), and fluorescence intensity was quantified using ImageJ software with subtraction of the background signal of five images per condition.

### 2.9. RNA Isolation and Quantitative PCR

Quantitative RT-PCR was used to verify siRNA inhibition. Total RNA was isolated from cells using the TRIzol method (Invitrogen). A total amount of 1 µg total RNA was used for DNAse-I treatment (amplification grade; Invitrogen) and reversed transcribed into first-strand cDNA using the Maxima First-Strand cDNA Synthesis Kit (Thermo Fischer Scientific). Primer sequences are as follows, *SULF2* forward 5′-TCCAAATCACTGGGACAACTGTGG-3′ and reverse 5′-TGCCTGTGCAGTCAGGTGATG-3′; *NRP2* forward 5′-GGAGCCCTGTGGTTGGATGTATG-3′ and reverse 5′-TCATCTGGAAACGTCCGGTCGT-3′. NCBI BLAST confirmed the specificity of the primers. The presence of a single PCR product was verified by both the presence of a single melting temperature peak and detection of a single band of the expected size on 3% agarose gels. Mean primer efficiencies as determined by LinReg [30] were nearly equivalent and ranged between 1.86 and 1.88. Non-template controls were included as a control. Real-time quantitative PCR was performed using a CFX96 real-time PCR detection system (Bio-Rad Laboratories, Hercules, CA, USA) [24]. PCR products that did not show a single melting temperature peak were excluded from analysis. Ct values were converted to arbitrary absolute amounts (2^−Ct^ × 1E12) and were expressed as a fold change compared to controls. Expression data were normalized to tyrosine 3-monooxygenase/tryptophan 5-monooxygenase activation protein zeta (YWHAZ) mRNA levels for each sample, forward 5′-ACTTTTGGTACATTGTGGCTTCAA-3′, and reverse 5′-CCGCCAGGACAAACCAGTAT-3′. Microarray data were obtained from a previous study [24].

### 2.10. Western Blots

HUVECs were transfected with si*SULF2* or siNT (as the control) as described above. After three days of incubation, the proteins were extracted using Pierce RIPA lysis and extraction buffer (Thermo Fisher Scientific), which was supplemented with a protease inhibitor cocktail (Roche Diagnostics, Almere, The Netherlands). Protein extraction was performed as per the manufacturer’s instructions, and the concentration was determined with the Bradford assay using Bradford reagent (Serva, Heidelberg, Germany) diluted in deionized water. Next, 20 µg of protein was separated on a 10% SDS-PAGE gel and then transferred overnight onto a nitrocellulose membrane (Whatman, Dassel, Germany) by wet blotting. The membrane was incubated for 1 h in Intercept TBS Blocking Buffer (Li-COR Biosciences, Homburg, Germany), which was diluted 1:1 with TBS. Next, the membrane was incubated overnight at 4 °C with the following primary antibodies: anti-NRP2 (1:1000; R&D) and β-actin as protein loading control (1:5000; Sigma–Aldrich); both antibodies were diluted in blocking buffer. On the following day, the membrane was washed three times with TBST (Tris-buffered saline, 0.1% Tween 20) and then incubated for 1 h at room temperature with secondary antibodies labelled with IRDye700 or IRDye800 (1:10.000; LI-COR Biosciences), which were diluted in TBST. Subsequently, the blot was washed twice with TBST and once with TBS before scanning. The blots were scanned using an Odyssey Imager (LI-COR Biosciences), and the density of the protein bands was quantified using ImageJ software. Experiments were performed in HUVECs isolated from three different donors.

### 2.11. Statistics and Data Correction

To correct for differences between donors, data from flow cytometry and spheroid experiments were corrected using factor corrections as described previously [31]. We assumed a normal distribution of our data, since outliers did not occur. For single column comparisons, an unpaired T-test was used, and for multiple comparisons, one-way ANOVA or two-way ANOVA tests were performed using GraphPad Prism version 8.0.0 for Windows (GraphPad Software, San Diego, CA, USA). Differences were considered statistically significant with a probability level of α < 0.05.

## 3. Results

### 3.1. Role of SULF2 in VEGFA_165_ and VEGFA_121_ Stimulation of HUVECs

**Figure 1:**
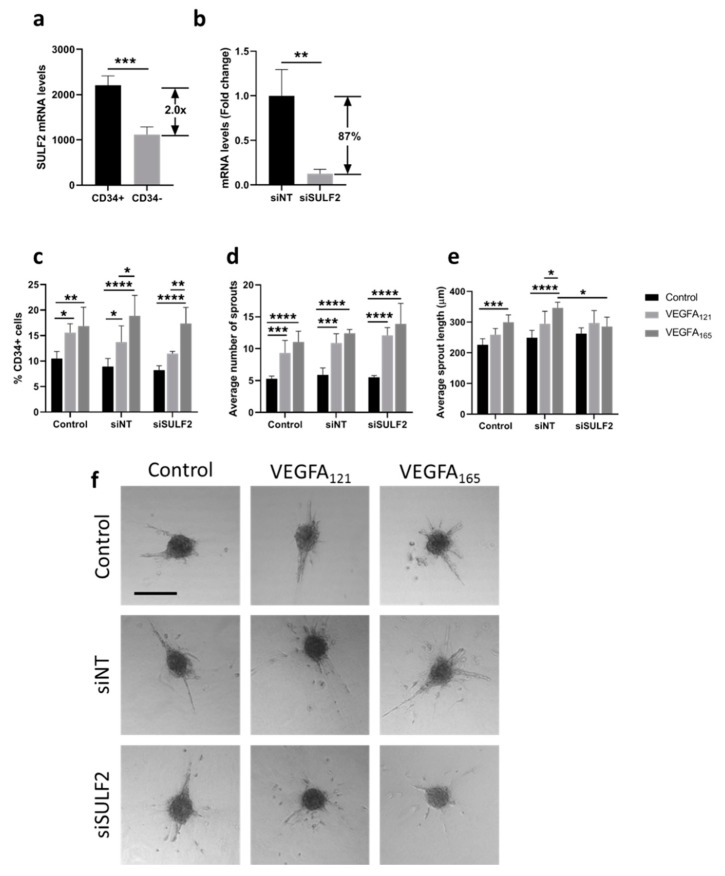
Effect of *SULF2* knockdown on VEGFA121- and VEGFA165-induced tip cell formation and sprouting. (**a**) Microarray analysis showed enrichment of *SULF2* mRNA in CD34^+^ cells as compared to CD34^-^ cells. HUVECs were treated with siNT, si*SULF2* or left untreated (Control) and were subsequently cultured in the presence or absence of VEGFA_165_ or VEGF_121_. (**b**) Treatment of cells with *siSULF2* resulted in a significant reduction in mRNA levels of 87% as compared to treatment with siNT. (**c**) Flow cytometric analysis of CD34^+^ cells (% of total number of cells). Average number (**d**) and length in µm (**e**) of sprouts per HUVEC spheroid. (**f**) Representative images of sprout formation from HUVEC spheroids. Results are presented as the mean ± standard deviation of three HUVEC donors. Unpaired T-tests (**a**,**b**), two-way ANOVA (**c**) or two-way mixed ANOVA (**d**,**e**) were used to calculate statistical differences (* *p* < 0.05, ** *p* < 0.01, *** *p* < 0.001, **** *p* < 0.0001). Scale bar = 250 µm.

**Figure 2:**
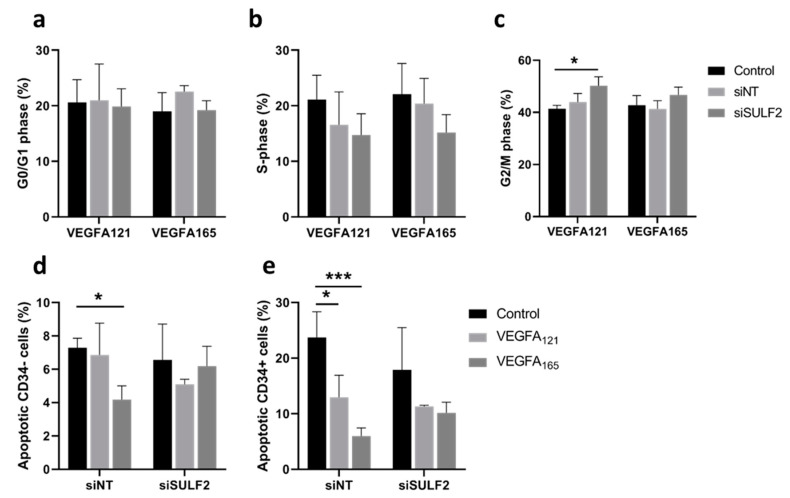
Effects of *SULF2* knockdown on VEGFA_121_- and VEGFA_165_-induced cell proliferation and apoptosis. HUVECs were cultured in the presence or absence of VEGFA_165_, VEGF_121_ or no VEGF (Control) after treatment with siNT or si*SULF2*. Flow cytometric analysis determined the percentage of cells in G0/G1 phase (**a**), S-phase (**b**), and G2/M phase (**c**). FACS analysis after annexin-5 and CD34 labeling determined the percentage of CD34^-^ non-tip cells (**d**) and CD34^+^ tip cells (**e**). Results are presented as the mean ± standard deviation of three HUVEC donors. Two-way ANOVA was used to calculate statistical differences (* *p* < 0.05, *** *p* < 0.001).

Differential binding of VEGFA splice variants to HSPGs on the endothelial cells or in the ECM may have an effect on downstream VEGF signaling, as VEGFA_165_ has an HS binding domain, whereas VEGFA_121_ does not [8]. SULF2 removes sulfate groups from HSPGs [16] and may therefore alter HS binding and mediate the bioavailability of these VEGFA variants. Previously published microarray data [24] showed that *SULF2* gene expression was twice as high in CD34^+^ tip cells than in CD34^-^ cells in vitro (Figure 1a). To investigate its possible role in VEGF-induced tip cell formation and angiogenic sprouting, we knocked down *SULF2* expression with siRNA. *SULF2* siRNA-mediated knockdown resulted in an 87% decrease in *SULF2* mRNA levels (Figure 1b). We were not able to verify *SULF2* knockdown at the protein level, since we could not find a working antibody for western blotting. The percentage of tip cells was increased after the addition of both VEGFA_121_ and VEGFA_165_, with only a marginal difference between VEGFA_121_ and VEGFA_165,_ and percentages were not altered by knockdown of *SULF2* (Figure 1c). In HUVEC spheroids, inhibition of *SULF2* did not affect the number of sprouts of spheroids in the presence of VEGFA_121_ or VEGFA_165_ (Figure 1d), but sprout length was reduced in the presence of VEGFA_165_ by 1.3-fold (*p* = 0.031) (Figure 1e). Treatment with VEGFA_165_ increased sprout length as compared to untreated spheroids under the control and siNT conditions to a higher extent than in the treatment with VEGFA_121_ under siNT conditions, but not after knockdown of *SULF2* (Figure 1e). This suggests that SULF2 has a role in VEGF-induced sprout elongation. Representative images of individual spheroids and their sprouts are shown in Figure 1f. Specific assays were performed to exclude major effects of apoptosis or proliferation confounding the final percentage of tip cells in these experiments. No significant differences in proliferation were found in HUVECs treated with *siSULF2* as compared to HUVECs treated with siNT (Figure 2a–c). Addition of VEGFA_165_ and, to a lesser extent, VEGFA_121_, reduced apoptosis in CD34^+^ tip cells but not in CD34^-^ HUVECs (Figure 2d,e). si*SULF2* treatment did not affect apoptosis in CD34^+^ and CD34^-^ HUVECs. Thus, although *SULF2* was preferentially expressed by tip cells, it does not seem to play a major role in their formation, survival or function as leading cells of sprouts.

### 3.2. NRP2 Is a VEGFR2 Co-Receptor Involved in VEGFA-Induced Sprouting, but Not in Tip Cell Formation

**Figure 3:**
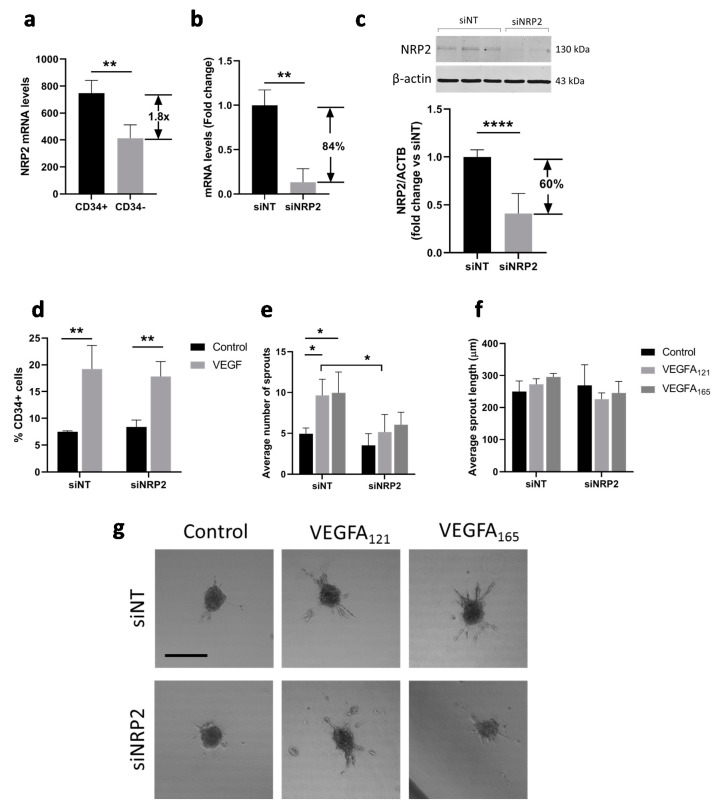
Effect of NRP2 knockdown on VEGF-induced tip cell formation and sprouting. (**a**) Microarray analysis showed enrichment of *NRP2* mRNA in CD34^+^ cells as compared to CD34^-^ cells. HUVECs were cultured in the presence or absence of VEGFA_121_ and/or VEGFA_165_ or after treatment with siNT or si*NRP2*; (**b**) Treatment of cells with si*NRP2* resulted in a significant reduction in mRNA levels of 84% as compared to treatment with siNT; (**c**) Treatment of cells with si*NRP2* resulted in a significant reduction in protein levels of 60% as compared to treatment with siNT. A representative western blot is shown. Quantification of NRP2 levels as compared to β-actin levels is shown below in three HUVEC donors; (**d**) Flow cytometric analysis of CD34^+^ cells (% of total number of cells). Average number (**e**) and length in µm (**f**) of sprouts per HUVEC spheroid; (**g**) Representative images of sprout formation from HUVEC spheroids. Results are presented as the mean ± standard deviation of three HUVEC donors. Unpaired T-tests (**a**,**b**) or Two-way ANOVA analyses (**c**–**e**) were used to calculate statistical differences (* *p* < 0.05, ** *p* < 0.01, **** *p* < 0.0001). Scale bar = 250 µm.

Neuropilin 1 (NRP1) and neuropilin 2 (NRP2) are co-receptors of VEGFR1 and VEGFR2 during VEGF signaling and were found to differentially bind VEGFA_121_ and VEGFA_165_, with VEGFA_121_ having a lower affinity for NRP1 and NRP2, suggesting a role of the SH-binding domain [5]. Both *NRP1* and *NRP2* mRNA are enriched in CD34^+^ tip cells as compared to CD34^−^ cells, but *NRP2* to a higher extent based on microarray and qPCR results [24]. In addition, no difference in mRNA levels was found for *NRP1* in CD34^+^ as compared to CD34^-^ hMVECs, whereas *NRP2* mRNA levels were significantly higher in CD34^+^ hMVECs [32]. We therefore specifically investigated the role of NRP2 in VEGF-induced tip cell formation and angiogenic sprouting. Previously published microarray data [24] revealed that *NRP2* mRNA levels were 1.8-fold higher in tip cells as compared to non-tip cells (Figure 3a). An efficient reduction of *NRP2* mRNA levels (Figure 3b) and protein levels (Figure 3c) was confirmed after siRNA-mediated knockdown of *NRP2*. Knockdown of *NRP2* did not affect the percentage of CD34^+^ tip cells in the presence or absence of VEGFA (Figure 3d). However, both the VEGFA_121_- and VEGFA_165_-induced increase in the number of sprouts was abolished after knockdown of *NRP2* (Figure 3e,g), but there was no change in sprout length after knockdown of *NRP2* in the presence or absence of VEGFA_121_ or VEGFA_165_ (Figure 3f,g). We found that NRP2, which is more strongly expressed by tip cells, does not seem to play a role in tip cell formation. However, as both VEGFA_121_ and VEGFA_165_ showed a diminished effect on sprout initiation in cells with reduced *NRP2* mRNA levels, our results suggest that both VEGFA splice variants depend on the presence of NRP2 for proper initiation of sprouting.

### 3.3. Role of SULF2 in NRP2 and VEGFR2 Protein Expression in Tip Cells and Non-Tip Cells

**Figure 4:**
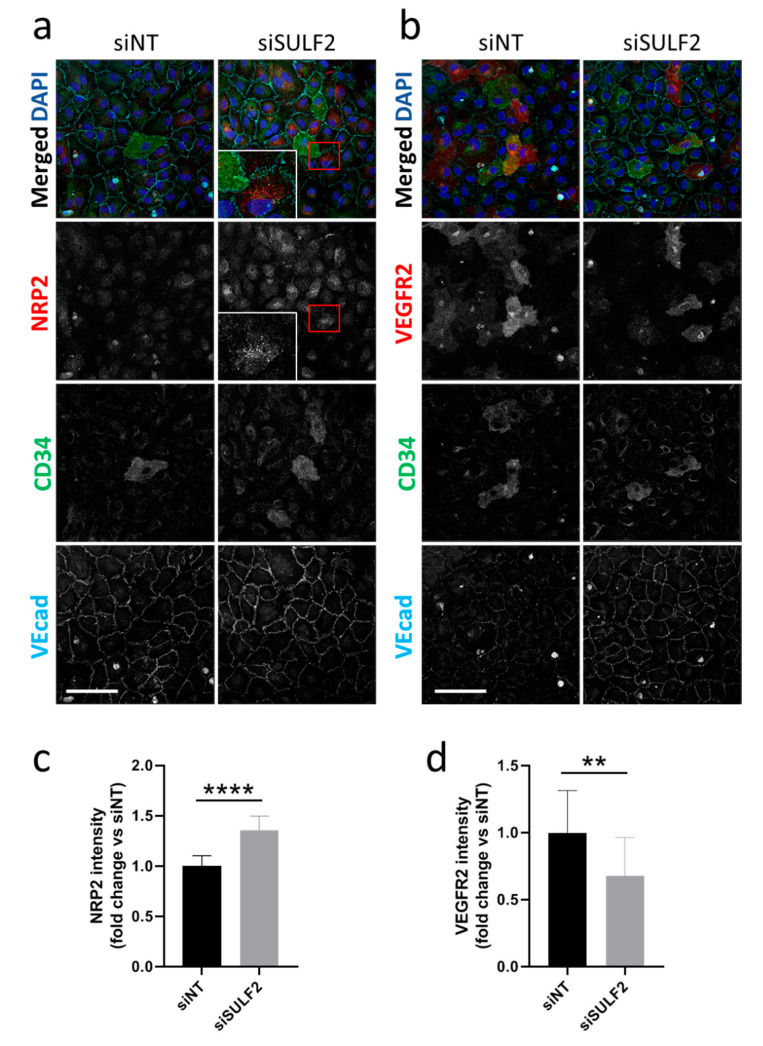
Changes in protein levels of NRP2 and VEGFR2 after *SULF2* knockdown. HUVECs were transfected with si*SULF2* or siNT as a control, seeded on coverslips and cultured for three days. After 2% PFA fixation, cells were stained with antibodies against (**a**) NRP2 or (**b**) VEGFR2 (red), CD34 (green), or VEcad (cyan) and with DAPI (blue). Representative images are shown for experiments on HUVECs of three donors. A 2.5-fold magnification of the area in the red box is presented. Scale bar = 50 µm. Quantification of NRP2 (**c**) and VEGFR2 (**d**) is shown below, relative to siNT with mean ± SD. ** *p* < 0.01, **** *p* < 0.0001.

To further investigate the role of SULF2 in VEGF-signaling in tip cells, we investigated the protein expression of NRP2 and VEGFR2 after *SULF2* knockdown. Protein expression of NRP2 was detected in all subtypes of HUVECs with no specific expression in CD34^+^ tip cells (Figure 4a). Knockdown of *SULF2* resulted in increased NRP2 levels by an average of 1.4-fold (*p* < 0.0001) in all cells (Figure 4a,b). NRP2 showed a punctuated staining (Figure 4a), suggesting localization in vesicles. VEGFR2 protein expression was predominantly found in tip cells, but to a lesser extent also in non-tip cells (Figure 4b). After *SULF2* knockdown, VEGFR2 was decreased in the total cell population by an average of 1.5-fold (*p* = 0.0065) (Figure 4d). These results suggests that SULF2 plays a regulatory role in endothelial expression of VEGFR2 and NRP2.

### 3.4. Role of SULF2 in VLDL Uptake by HUVECs

**Figure 5:**
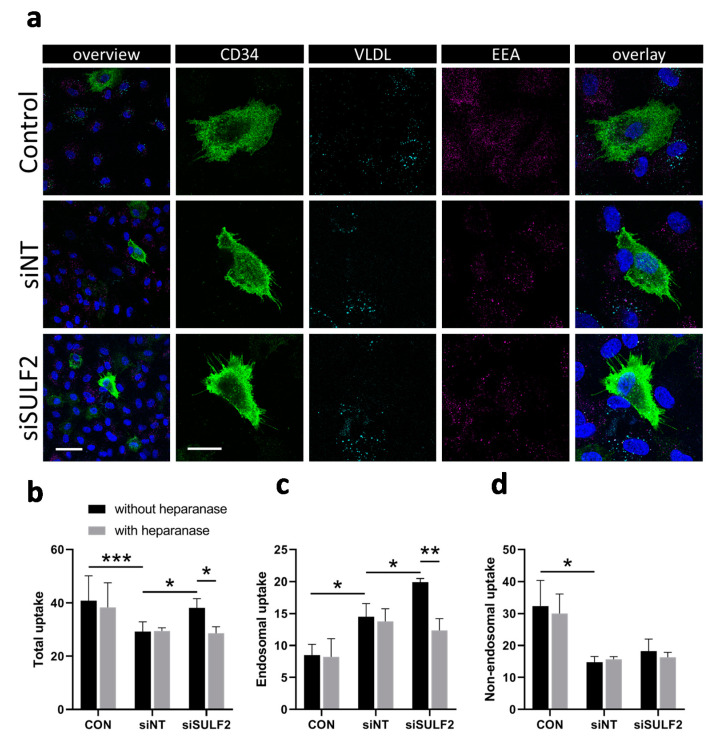
Knockdown of *SULF2* leads to increased uptake of VLDL in non-tip cells. (**a**) Demonstration of endosomal uptake of fluorescently-labeled VLDL by CD34^-^ but not CD34^+^ cells. Staining of CD34 (tip cells, green), VLDL (cyan), endosomes (EEA, magenta), and nuclei (DAPI, blue) in HUVECs and overlaid images at low (bar = 50 µm) and higher (bar = 20 µm) magnification. (**b**–**d**) Quantification of total, endosomal, and non-endosomal uptake of fluorescently-labeled VLDL by HUVECs after siRNA-mediated knockdown of *SULF2*. Heparanase was used as a control as it removes all HS chains from HSPG proteins. Cells were stained with DAPI and EEA1 for nuclei and endosomes. The numbers of VLDL particles per cell were counted using Matlab and overlap with EEA1 staining was considered to indicate endosomal uptake. Students*’ t*-tests were used to calculate statistical differences (* *p* < 0.05, ** *p* < 0.01, *** *p* < 0.001) using HUVECs of three donors and at least seven cells per condition in each experiment.

HSPGs have a role in VLDL uptake by ECs, which is reduced by SULF2 [18]. We further investigated the role of SULF2 in HSPG-mediated VLDL uptake by analyzing fluorescently-labeled VLDL in endosomes in HUVEC cultures (Figure 5). VLDL uptake occurred mainly in CD34^-^ non-tip cells rather than in CD34^+^ tip cells (Figure 5a). Next, we investigated whether SULF2 is important for VLDL uptake by CD34^−^ cells. Figure 5b–d shows that si*SULF2* treatment stimulated endosomal but not non-endosomal uptake of VLDL in non-tip cell HUVECs. The effect of si*SULF2* treatment was annihilated by heparanase activity, an enzyme which cleaves all HS molecules. These findings confirmed that SULF2 inhibits endosomal VLDL uptake and show that this process is specific for CD34^-^ cells in vitro.

### 3.5. VLDL Uptake Does not Affect the Tip Cell Percentage but Enhances the Number of Sprouts

**Figure 6:**
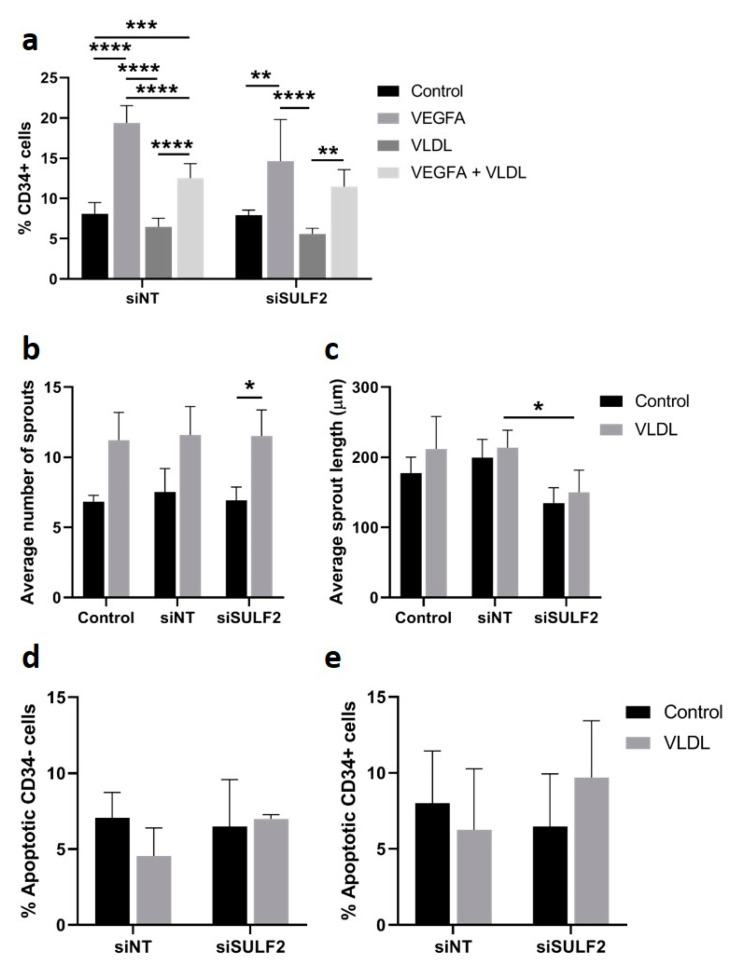
Effect of VLDL on tip cell formation, sprouting, and apoptosis. HUVECs were cultured in the presence or absence of VEGFA_165_ and/or VLDL or after treatment with siNT or si*SULF2*. (**a**) Flow cytometric analysis of CD34^+^ cells (% of total number of cells). Average number (**b**) and length in µm (**c**) of sprouts per HUVEC spheroid. Relative amount of apoptotic CD34^-^ (**d**) and CD34^+^ cells (**e**). Results are presented as the mean ± standard deviation of three HUVEC donors. Two-way ANOVA was used to calculate statistical differences (* *p* < 0.05, ** *p* < 0.01, *** *p* < 0.001, **** *p* < 0.0001).

Specific VLDL uptake by CD34^-^ cells may be explained by its role in angiogenesis as a provider of biomass for proliferating stalk cells [33]. We tested the possible consequences of inhibition of VLDL uptake on the percentage tip cells and on vessel sprouting in the spheroid model. The addition of VLDL did not have an effect on the percentage of tip cells under control conditions, and there was a modest but significant inhibitory effect of exogenous VLDL on VEGFA-induced tip cell formation after treatment with siNT (Figure 6a). In cells treated with *siSULF2*, exogenous VLDL did not have an effect on the percentage of tip cells. The addition of VLDL to HUVEC spheroids resulted in an increased number of sprouts per spheroid (Figure 6b), but did not change the sprout length (Figure 6c), whereas *siSULF2* inhibited sprout length to some extent (1.4-fold, *p* = 0.016) (Figure 6c). Apoptosis levels of CD34^-^ or CD34^+^ cells were not changed upon addition of VLDL or knockdown of *SULF2* (Figure 6d,e). Taken together, we found that exogenous VLDL induces sprouting in cultured HUVECs, but does not induce tip cell formation.

## 4. Discussion

In the present study, we investigated whether the differential HSPG-binding capacity of VEGFA splice variants or the presence of VEGFR2 co-receptors NRP1 and NRP2 regulates tip cell formation and sprouting angiogenesis. To this end, we experimentally inhibited expression of *SULF2*, an enzyme that selectively removes 6-O sulfate groups from HS chains on HSPG molecules and which has high expression in tip cells. We found that VEGFA_165_ and VEGFA_121_ had similar effects on tip cell formation and angiogenesis in vitro under conditions with or without reduced *SULF2* expression. Furthermore, knockdown of *SULF2* only modestly reduced sprout length in spheroids and did not affect the number of sprouts in spheroids or tip cell formation in HUVEC cultures. This suggests that differential HS binding of VEGFA splice variants does not play a major role in tip cell formation and sprouting angiogenesis in vitro. However, we observed that uptake of VLDL occurred only in non-tip cells, was increased upon knockdown of *SULF2,* and induced sprouting but not tip cell formation. Finally, sprout initiation by VEGFA was affected by the presence of the VEGFR2 co-receptor NRP2.

VEGFA is the main driving force for sprouting angiogenesis [2]. Its binding to VEGFR2 induces a cascade of signaling processes resulting in increased migration of tip cells, increased proliferation of stalk cells [24,34], and inhibition of apoptosis in both tip and stalk cells [2,3]. We show here that both VEGFA_165_ and VEGFA_121_ consistently induced sprouting and CD34^+^ tip cell formation, whereas VEGFA_121_ had a slightly lesser effect on sprout length growing from endothelial spheroids in vitro. One of the main differences between VEGFA_165_ and VEGFA_121_ is HS binding. It has been reported that binding of VEGFA_165_ to extracellular HSPGs alters the activation of downstream signaling pathways in ECs in vitro: extracellular protein binding of VEGFA_165_ caused elevated activation of AKT serine/threonine kinase 1 (AKT), whereas unbound VEGFA_165_ mainly activated p38 [35]. Activation of p38 induces migratory signals, whereas activated AKT causes proliferative and survival signals in ECs [36,37,38]. The migratory signaling cascade is relevant for tip cells during angiogenesis, whereas the latter signaling cascade is relevant for stalk cells. Moreover, excessive amounts of 6-O sulfate groups on HSPGs have been shown to reduce binding of VEGFA to VEGFR2 [14]. As SULF2 removes 6-O sulfate groups, knockdown of *SULF2* was expected to result in an excess of 6-O sulfate groups on HSPGs and reduced sprouting. This is in agreement with the results shown in Figure 1e and Figure 6c, which suggest that binding to HSPGs by VEGFA_165_ has a modest enhancing effect on sprout elongation. In transgenic mice expressing only VEGFA_121_, which does not bind to HSPGs, this may also be the underlying mechanism of disturbed vascular development leading to irregular vascular networks [8].

Binding of the VEGFR2 co-receptor NRP2 only affected VEGFA-induced sprouting in vitro, and not tip cell formation. In the literature, NRP2 has mainly been associated with EC survival [39] and angiogenesis [40], but has been less studied than its family member NRP1, which is involved in tip cell regulation during angiogenesis and in neuronal development [41,42]. Although NRP2 is mainly associated with lymphatic expression and has a role as a co-receptor for VEGFR3 in lymphangiogenesis [43], NRP2 expression is also found in tip cells during angiogenic sprouting [44]. NRP1 and NRP2 have been shown to bind VEGFA_165_, but whether they also bind VEGFA_121_ has been a subject of debate [5,45,46]. Since we found that *NRP2* mRNA is mainly expressed in CD34^+^ tip cells, it may play a role in tip cells during initiation of sprouting. Here, we show that knockdown of *NRP2* affects VEGF_121_- as well as VEGF_165_-induced sprouting, indicating that there is a functional interaction between NRP2 and both splice variants without preference for one or the other. In addition, expression of the NRP2 protein was not specific for tip cells in vitro. Altogether, it seems that NRP2 is not involved in induction of the tip cell phenotype by VEGFA but supports the formation of new vessel sprouts by VEGFA in another manner.

Besides altering growth factor binding to the ECM, SULF2 also regulates uptake of VLDL particles by endothelial cells [17]. VLDLs are triglyceride-rich particles that provide fatty acids that are necessary for cell proliferation [47,48] and thus are important for sprout elongation by proliferating stalk cells. Angiogenesis is mainly driven by glycolysis [49,50,51]. Tip cells have a flexible energy metabolism, which fits their role as pioneers, moving into tissues during angiogenesis, where the availability of substrates and oxygen may vary greatly [50,51]. We show here that tip cells do not take up VLDL particles, whereas non-tip cells do. This process may be regulated by SULF2-induced alteration of HSPGs, as was shown by abolition of these effects by heparanase which removes all extracellular HS chains [7]. Our in vitro experiments show that VLDL itself affects sprouting: addition of exogenous VLDL did not induce stalk cell proliferation or sprout elongation, but it increased the number of sprouts. This is consistent with previous studies which have shown that VLDL can elicit activation of several pro-angiogenic pathways such as PI3K and PPAR-γ [23,52]. The inhibitory effects on VEGF-induced tip cell formation seem contradictory in this case, but may be the result of a reduced number of HSPG molecules on the cell surface because they are endocytosed along with the VLDL molecules [53]. We studied the effects of exogenous VLDL only. Free fatty acids (FFA) are essential for proliferating cells, as has been extensively discussed by Carmeliet et al. [54]. Not only are FFA degraded to provide energy, they also provide biomass for dNTP synthesis needed for DNA replication in proliferating cells. We hypothesize that when VLDL cannot enter cells, the cells can generate FFA by de novo lipogenesis or, more likely, switch their metabolism and use glucose instead as energy source.

Our study is limited because the in vitro environment may differ in unknown aspects from angiogenic sprouting in vivo. In our experiments, HUVECs were seeded on gelatin-coated plastic and were covered by medium supplemented with VEGFA or VLDL which was probably mainly in contact with the ECs on the side of the medium, which probably has similarities to the luminal side of endothelium in vivo. During sprouting in vivo, tip cells, which lack a luminal side, basal lamina or glycocalyx, penetrate the ECM, while stalk and phalanx cells produce and are surrounded by a basal lamina. The ECM consists of a large number of proteins, including HSPGs. In our experiments, we could not discriminate between the effects of our interventions on HSPGs in the glycocalyx of the cultured ECs [12], which is present in vivo on the luminal side [10,55], versus HSPGs in the ECM, i.e., in the basal lamina deposited by the cells on the plastic. Nevertheless, since we have shown that expression of *SULF2* was stronger in CD34^+^ tip cells compared to CD34^-^ cells, a finding recently reported by others [56], sulfation of HSPGs in the ECM may well play a regulatory role in vessel sprouting and maturation during angiogenesis in vivo.

## 5. Conclusions

On the basis of our experiments using VEGFA splice variants with or without HS-binding domains and knockdown of *SULF2*, an enzyme that removes sulfate groups from HSPGs, we conclude that HSPG-binding of VEGFA may have a small role in the elongation of angiogenic sprouts but does not affect the initiation of the sprouts or tip cell formation. VLDL uptake, on the other hand, is regulated by HSPG-binding, as *SULF2* mRNA knockdown increased VLDL uptake. Furthermore, uptake of VLDL was limited to non-tip cells and induced sprouting but not tip cell formation. This suggests that VLDL has a role in sprouting, by delivering fatty acids necessary for stalk cell proliferation. Finally, we showed that the VEGFA–VEGFR2 co-receptor NRP2 plays a role in sprout initiation by tip cells.

When we consider our results in the context of sprouting angiogenesis in vivo, they seem to fit well with previously reported results. Tip cells lack a basal lamina and direct contact with HSPGs, and are therefore more likely to bind unbound VEGFA_165_ (or VEGFA_121_), which activates p38 [35], inducing the migratory signals typical of these cells. The high expression of SULF2 on tip cells that we report here may have a function in removing 6-O sulfate groups on any HSPG present in the ECM, enhancing the binding of VEGFA to VEGFR2 [14] and/or suppressing VLDL uptake in these cells. In contrast, stalk cells, which have a basal lamina, are more likely to encounter VEGFA_165_ bound to HSPGs, causing elevated activation of AKT serine/threonine kinase 1 (AKT) and subsequent proliferative and survival signals needed by these cells [36,37,38]. The latter mechanism may also explain the slightly lesser effect of VEGFA_121_ compared to VEGFA_165_ on the length of sprouts growing from endothelial spheroids in vitro as observed in this study. In addition, low expression of SULF2 in stalk cells allows uptake of VLDL, necessary for the proliferation of these cells.

## Data Availability

Not applicable.
